# Diurnal variation of salivary oxidative stress marker 8-hydroxyguanine

**DOI:** 10.1186/s41021-019-0138-3

**Published:** 2019-12-10

**Authors:** Sintaroo Watanabe, Yuya Kawasaki, Kazuaki Kawai

**Affiliations:** 1 Department of Environmental Oncology, Institute of Industrial Ecological Sciences, University of Occupational and Environmental Health, Japan, 1-1 Iseigaoka, Yahatanishi-ku, Kitakyushu, 807-8555 Japan; 2 Japan Marine United Corporation Kure Shipyard, 2-1 Showa-cho, Kure-shi, 737-0027 Japan

**Keywords:** Oxidative stress, 8-hydroxyguanine (8-OHGua), Saliva, Diurnal variation

## Abstract

**Introduction:**

Oxidative stress is a risk factor for life-style related diseases, including cancer. We recently reported that the oxidative stress marker 8-hydroxyguanine (8-OHGua) can be measured in saliva non-invasively. Understanding the diurnal pattern of salivary 8-OHGua levels is crucial for evaluating the oxidative stress. In this study, we analyzed the diurnal variation of salivary 8-OHGua levels.

**Findings:**

The salivary 8-OHGua levels were relatively stable in the daytime (10:00–22:00). The daytime 8-OHGua levels seemed to represent the individual oxidative stress status. The average amount and the variation of the salivary 8-OHGua levels immediately after awakening were higher than those of the daytime levels.

**Conclusions:**

The 8-OHGua levels in saliva exhibited diurnal variation. The levels were higher at the time of awakening. At this point, the daytime levels of salivary 8-OHGua may be appropriate for evaluating the individual oxidative stress status. Further study is needed for understanding and utilizing the 8-OHGua levels at the time of awakening.

## Introduction

Oxidative stress is a known risk factor for lifestyle-related diseases, such as cancer [1, 2]. As a representative oxidative stress marker, 8-hydroxy-2′-deoxyguanosine (8-OHdG), an oxidative damage marker for nucleobases, has been widely analyzed using urine as a specimen. Urinary 8-OHdG measurement and oxidative stress evaluation may contribute to the prevention of lifestyle-related diseases. In addition to urinary 8-OHdG, we have found that salivary 8-hydroxyguanine (8-OHGua) might be useful as a new oxidative stress marker [3], because saliva can be collected more easily than urine. On the other hand, some salivary biomarkers, such as cortisol [4] and alpha-amylase [5, 6], show diurnal variations. Grew et al. [7] reported that the urinary 8-OHdG levels did not show diurnal variation. However, little is known about the diurnal variation of salivary 8-OHGua. In order to use salivary 8-OHGua as an oxidative stress biomarker, it is crucial to establish its diurnal pattern. The aim of the present study is to investigate the diurnal variation of salivary 8-OHGua and examine the appropriate time of saliva collection for the oxidative stress evaluation.

## Materials and methods

### Saliva collection

Ten minutes after rinsing the month with water, 2 mL of saliva were directly collected into a polypropylene tube from 6 subjects (4 male and 2 female), aged 20–60. The collected saliva samples were frozen at − 20 °C until analysis. At the time of analysis, a 300 μL portion of thawed saliva was mixed with 15 μL of proteinase K (Wako Pure Chemicals, Tokyo, Japan), at a concentration of 20 mg/mL. The mixture was incubated at 37 °C for 1 h. After drying with a vacuum centrifuge for 12 h, 300 μL of diluent (1.8% acetonitrile, 62 mM sodium acetate, 0.01 mM sulfuric acid) was added. The samples were clarified with a centrifugal filter (Amicon Ultra Ultracel-10 K, Merk Millipore Ltd., Darmstadt, Germany). The 8-OHGua level was analyzed with an HPLC-ECD system, using 20 μL of the filtrate as the sample. The 8-OHGua level was analyzed with a column switching HPLC-ECD system, using 20 μL of the filtrate as the sample [3]. The HPLC was equipped with an anion exchange column (MCI GEL CA08F, 7 μm, 1.5 × 30 mm + 1.5 × 90 mm, solvent A, 60 μL/min) and reverse phase column (GL Sciences, InertSustain C18, 3 μm, 4.6 × 250 mm, solvent B, 0.6 mL/min). Solvent A was 2% acetonitrile in 0.3 mM sulfuric acid, and solvent B was 9 mM K_2_HPO_4_, 25 mM KH_2_PO_4_, 0.5 mM EDTA•2Na, and 2.5% acetonitrile. According to the 8-OHGua standard solution, the coefficient of variation of the 8-OHGua level was within 5%.

### Statistical analyses

For statistical analysis, EZR (Jichi Medical University Saitama Medical Center Management, free software) was used [8]. The salivary 8-OHGua levels of each individual and the 6 subjects together were investigated at each collection time by the Friedman test. The statistical significance level was set at *p* < 0.05.

## Results

**Fig. 1:**
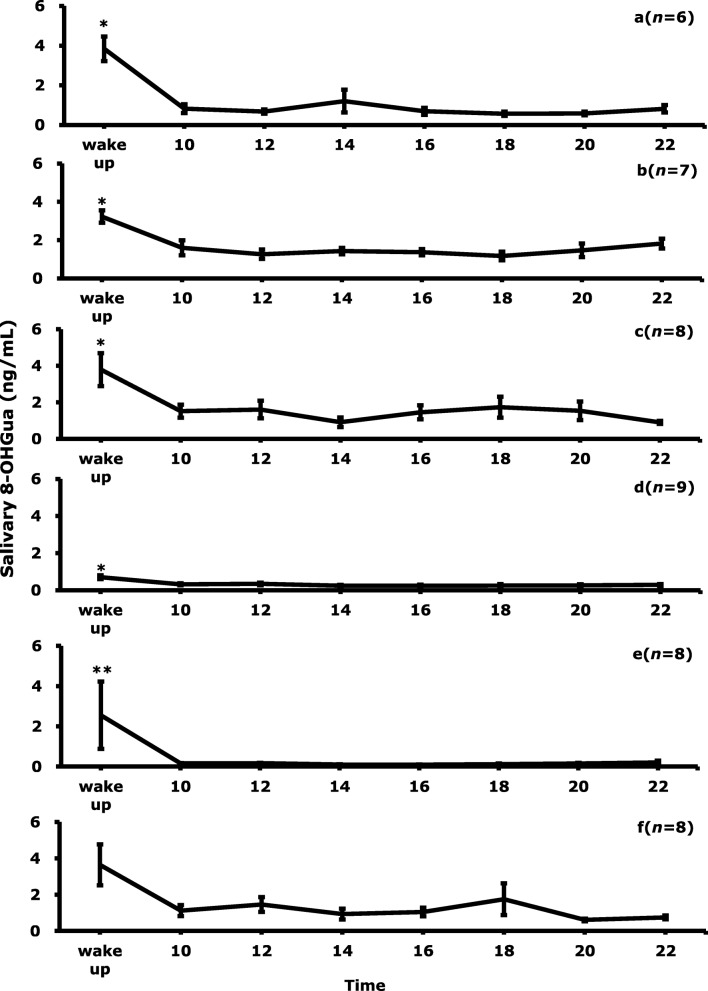
Diurnal variation of salivary 8-OHGua levels for each subject. **a**: 40s female, **b**: 60s female, **c:** 20s male**, d**: 30s male, **e**: 50s male, **f**: 60s male. Bars are mean ± SE, *n*: number of saliva collections, ^*^p < 0.05, ^**^p < 0.01, by Friedman test

**Fig. 2:**
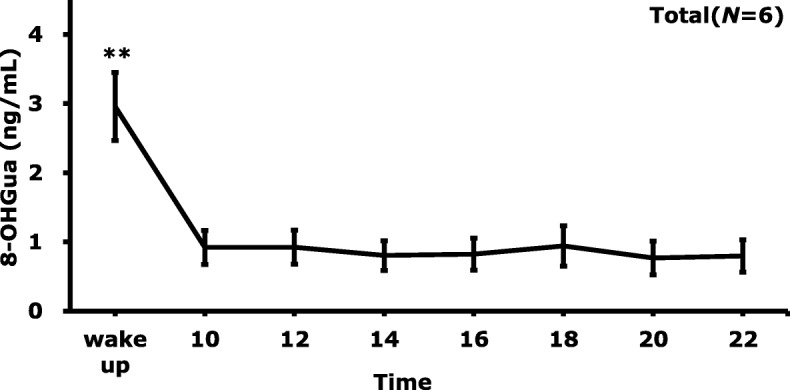
Diurnal variation of salivary 8-OHGua levels for all subjects (*N* = 6). Bars are mean ± SE, ^**^p < 0.01, by Friedman test

The diurnal changes of salivary 8-OHGua levels at the time of awakening and every 2 h, from 10:00 to 22:00, are shown in Fig. 1. Each point represents the mean level of 6–9 samples collected on different days. The salivary 8-OHGua levels were significantly higher at the time of awaking, as compared to the other time points. There were no significant differences in the 8-OHGua levels in saliva between 10:00 and 22:00. It seems that each person has an individual characteristic value, except for at the time of awakening. Although the number of subjects was limited, the diurnal profile of 8-OHGua did not differ between male and female. The diurnal variation of the salivary 8-OHGua levels for all subjects is shown in Fig. 2. The average level of salivary 8-OHGua immediately after awakening was about three-fold higher than that of the other time periods. In addition, the standard error of the salivary 8-OHGua levels was about twice as high at the time of awakening, as compared to the case of the samples obtained at the other time periods.

## Discussion

The present study revealed that the salivary 8-OHGua level upon awakening was higher than those during the daytime, and remained relatively stable during the day. Some salivary biomarkers show circadian variations. For example, the salivary α-amylase activity is reportedly low from night to dawn and high during the day [6, 9]. In contrast, the levels of melatonin [10], cortisol [4], chromogranin A (CgA) [4], and secretory globulin A (sIgA) [11] in saliva were high from night to dawn and low during the day. Salivary cortisol [12], one of the potential biomarkers for mental stress, showed similar diurnal variations to the 8-OHGua. Salivary 8-OHGua would be expected as a potential stress marker in industrial settings. Although the detailed mechanism remains unknown, the involvement of clock genes was suggested as the mechanism underlying the diurnal variation of the melatonin and sIgA levels. The sympathetic nerve activity was also suggested as the mechanism for the diurnal variation of the CgA levels. In addition, the salivary flow rate was involved in the diurnal variation of the secretion of antidiuretic hormone (ADH), because the secretion of ADH is increased at night and ADH suppresses salivary secretion [13]. In this study, although the flow rate of the saliva was not measured at each sampling point, the subjects did not report any difference in the flow rate in the morning, as compared to the rest of the day. The effect of the salivary flow rate on the 8-OHGua level may be limited. The higher amount of salivary 8-OHGua at the time of awakening may reflect a causal effect of the characteristic diurnal variation of saliva. Previous analyses revealed that the other oxidative stress marker, urinary 8-OHdG, does not show diurnal variation [7]. The subjects rinsed their mouths before saliva collection, but the samples could still be contaminated with a small amount of blood [14]. This might also affect the salivary 8-OHGua level. To confirm the value of the salivary 8-OHGua as an oxidative stress marker, future comparisons with other oxidative stress markers, such as urinary 8-OHdG, will be made with the same subject. Recently, we reported that the salivary 8-OHGua levels were significantly elevated with age, smoking, hypertension, and excess visceral fat [15]. In the report, saliva samples were collected in the morning (8 a.m. - 11 a.m.). It may be more appropriate to collect the saliva during the day, for evaluations of the salivary 8-OHGua levels to assess the oxidative stress status. Further examination is needed to reveal the meaning of the higher level and of salivary 8-OHGua at the time of awakening.

## Data Availability

All data generated or analyzed during this study are included in this published article.

## Abbreviations

8-OHdG: 8-hydroxy-2′-deoxyguanosine
8-OHGua: 8-hydroxyguanine
ADH: antidiuretic hormone
CgA: chromogranin A
ECD: electrochemical detector
sIgA: secretory immunoglobulin A
